# Study on vibration characteristics of roadheader cutting pre-cracked hard rock

**DOI:** 10.1038/s41598-026-37089-7

**Published:** 2026-01-21

**Authors:** Hongmei Liu, Faxu Li, Jingqiang He, Wenlong Wang, Haijian Zhang

**Affiliations:** 1 School of Mechanical Engineering, Liaoning Technical University, Fuxin, 123000 China; 2 CCTEG Times Power (Taiyuan) Co., Ltd., Taiyuan, 030032 China

**Keywords:** Hard rock boring, Roadheader, Mechanical pre-cracking, DEM–MFBD two-way coupling, Vibration characteristics, Energy science and technology, Engineering

## Abstract

To address severe equipment vibration and large load fluctuations during hard-rock excavation, this study proposes mechanical pre-cracking as a preparatory treatment. A hard-rock model containing pre-cracked holes is developed using the discrete element method; the crack initiation and propagation induced by a hydraulic cracker are simulated. The rock models before and after pre-cracking and a rigid–flexible coupling model of the roadheader are then analysed jointly via DEM–MFBD two-way coupling, and the cutting loads and vibration responses are systematically examined. Results indicate that the three-way average load on the cutting head is reduced by 8.2% and the load fluctuation coefficient decreases from 0.0242 to 0.0213 following rock pre-cracking, effectively mitigating impact loads. Frequency-domain analysis shows that, within the principal vibration band of 20–30 Hz, the vibration-acceleration amplitudes of the cutting head, cutting arm and slewing table are reduced by 14.7%, 8.7% and 3.6%, respectively, demonstrating a “near-loaded component” vibration response. This reveals an attenuation law along the transmission path: components closer to the load exhibit superior vibration damping compared with remote elements. The study confirms that mechanical pre-cracking achieves effective vibration attenuation at the source by reducing the overall stiffness of the rock mass and altering the rock-breaking pattern, thereby providing a theoretical basis and engineering reference for improving the efficiency and reliability of hard-rock tunnelling equipment.

**Supplementary Information:**

The online version contains supplementary material available at 10.1038/s41598-026-37089-7.

## Introduction

With the advancement of deep mineral-resource development and tunnelling projects, hard-rock excavation increasingly confronts technical challenges such as markedly higher rock strength and greater difficulty in rock breakage. Conventional mechanical rock-breaking methods (e.g., roadheader cutting) suffer from high energy consumption, rapid tool wear and severe vibration, which substantially limit construction efficiency and equipment reliability. Contemporary practices increasingly adopt cracking-assisted rock-breaking techniques (for example, upward cracking) to improve hard-rock boring^1^. Mechanical pre-cracking leverages the rock’s comparatively low tensile strength and its propensity for tensile damage to effect controlled rock splitting and crack initiation.

Considerable systematic research has been devoted to the mechanics and propagation laws of pre-existing cracks. M. Eftekhari et al.^2^ experimentally examined crack propagation in specimens with diagonal cracks under uniaxial compression and reported that crack propagation deviates from the original orientation, extending from the crack tip toward the loading plate. Zhou et al.^3^ investigated the influence of peripheral pressure loading mode, cracking-hole location and free surfaces on crack development, finding that peripheral pressure applied in the rising direction inhibits crack initiation and growth, whereas pressure applied parallel to the rising direction promotes crack propagation. Arshadnejad et al.^4^ developed empirical and finite-element models for the arrangement of air holes to study the effect of hole spacing on crack propagation in rock materials. Song Dao et al.^5^ employed a cohesive zone method to simulate upheaval cracking, correlating upheaval force characteristics with crack growth and energy evolution; they concluded that macroscopic fracture occurs when the upheaval force reaches its peak, after which the upheaval force decreases as rock strength diminishes. Zai Penghui^6^ combined laboratory tests, numerical simulation and theoretical analysis to study single- and multi-hole rupture behaviour in rocks containing prefabricated cracks, showing that fracture initiation pressure in specimens containing prefabricated cracks decreased by more than 53% compared with intact specimens; good agreement was observed among theoretical models, numerical simulations and test data. Under multi-hole conditions, the pressure required for crack penetration increases with hole spacing and peripheral pressure; however, the rate of decrease in penetration pressure slows with rising peripheral pressure and is not markedly influenced by changes in hole spacing. Wang J. et al.^7^ simulated crack propagation by constructing a continuous–discontinuous unit method that employs unit rupture modes; their results demonstrate that the method can visualise the entire crack evolution process from nucleation and extension to penetration and eventual formation of macroscopic fractures.

Research on the vibration characteristics of roadheaders has predominantly followed two methodological routes. The first route conducts vibration simulation of the whole machine or its components using multi-body dynamics. For example, Zhao Lijuan et al.^8^ prepared a transient load programme for the interceptor head in MATLAB to calculate impact loads under transverse pendulum conditions; these loads were subsequently imported into a longitudinal-axis roadheader rigid–flexible coupling vibration model for forced-vibration analysis, yielding the principal modal parameters and the system’s susceptible vibration patterns. Wang Junliang^9^ examined the vibration response of a longitudinal-axis roadheader under random excitation using the Lagrange equation with a virtual excitation approach; results indicated that, under external random excitation, the transverse displacement amplitudes of the cutting head and cantilever are slightly lower than those in the longitudinal direction, while the transverse displacement of the body is significantly smaller than its longitudinal counterpart. Zhang Zhenshan et al.^10^ performed finite-element simulations of the vibration-fatigue characteristics of a roadheader slewing table using ANSYS coupled with the Palmgren–Miner fatigue criterion; their findings indicate that X-axial vibration is the principal contributor to slewing-table fatigue damage, and that targeted vibration damping can significantly extend overall life. Minjun Zhang et al.^11^ built a finite-element model of the slewing table using Pro/E and ADAMS to analyse vibration under different working conditions and validated the method by comparing simulation and experimental results. The second route adopts structural modal and vibration analysis based on finite-element techniques. For example, Wang Dongdong^12^ used finite-element simulation on an EBG200B roadheader to investigate modal vibration patterns of the cutting structure, identifying intrinsic frequencies and locations of maximum amplitude in operational environments to support cutting-part design optimisation and damping strategies. Liu Yong et al.^13^ modelled the three-dimensional assembly and finite-element dynamic response of an impact rock-breaker mechanism; modal analysis identified four typical positions for dynamic response analysis under extreme operating attitudes, and frequency–amplitude response characteristics in the x, y and z directions were obtained. Their results show significant vibration peaks near 4 Hz, 38 Hz and 48 Hz—corresponding to the first two and the sixth–seventh orders of the mechanism’s natural frequencies—while amplitudes remain small and responses stay smooth across the remaining frequency range.

Despite the advances above, notable limitations remain. Most existing studies treat rock-wall pre-cracking and roadheader cutting as independent systems and therefore do not systematically investigate the pre-cracked rock mass as the actual cutting target. Methodologically, although software such as ADAMS and ANSYS is commonly employed, these tools alone cannot fully capture the two-way interaction between rock fragmentation and mechanical dynamics during cutting; in particular, two-way coupled simulations that integrate discrete-element models with rigid–flexible multibody dynamics are scarce. To address this gap, the present study constructs a rock-wall model based on discrete-element principles and mechanical pre-cracking technology, and employs two-way coupling between the discrete-element software EDEM and the multibody-dynamics package RecurDyn to simulate cutting of rock walls before and after pre-cracking. The vibration characteristics of the roadheader under both conditions are analysed and compared quantitatively to assess the vibration-suppression effect afforded by pre-cracking.

## Principle of mechanical pre-cracking technology

**Fig. 1:**
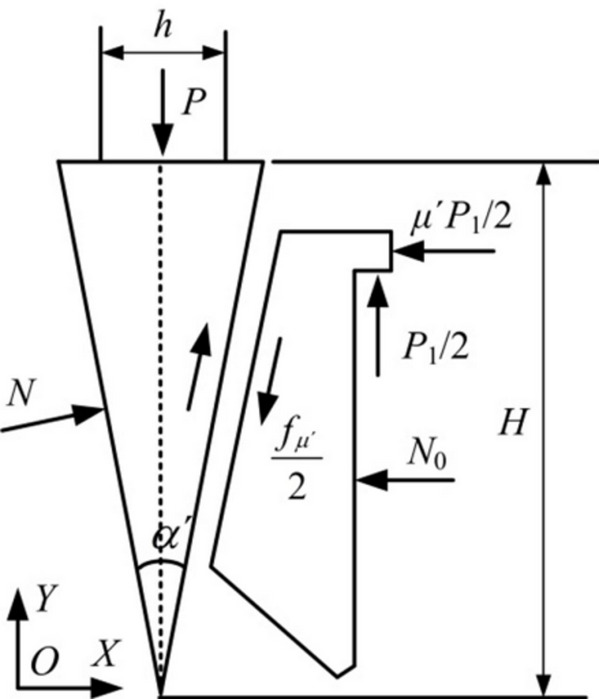
Force analysis of wedge.

Mechanical pre-fracturing is an efficient rock-breaking method that exploits the mechanical characteristic that rock tensile strength is far lower than its compressive strength. The implementation procedure comprises: first, prefabricating drill holes in the rock mass; subsequently inserting a hydraulic cracker into these holes. During operation, the hydraulic system drives the wedge axially, and the axial thrust is converted, by the tip-splitting structure, into a radial static cracking force acting on the hole wall. This force generates highly concentrated tensile stresses around the borehole; when the tensile stress exceeds the rock tensile strength, cracks initiate at the hole wall and propagate along a predetermined direction, ultimately achieving controllable and directional fracturing of the rock mass. The force analysis of the wedge is shown in Fig. 1, and the mechanical relation can be expressed by the following equation:

1 $$P = \frac{{\pi d^{2} p}}{4} = 2N\left( {\frac{h}{{2H}} + \mu ^{\prime}} \right)$$

Style: *P* — axial thrust output from the hydraulic cylinder, N; *d* — hydraulic cylinder diameter, cm; *p* — working oil pressure, MPa; *N* — radial pre-cracking force acting on the drilled hole wall, N; *h/H*— wedge travel as a function of ramp length; *µ’*— coefficient of friction between the wedge and the splitting wedge.

Compared with conventional mechanical rock-breaking methods, the principal advantage of mechanical pre-cracking is that it changes the rock-breaking mechanism: by applying continuous static tensile stress to the rock mass, the rock is efficiently fractured according to its inherent low tensile strength. This approach fundamentally mitigates the issues associated with traditional cutting or impact methods—intense tool wear, high vibration and elevated specific energy consumption—and demonstrates superior economy and reliability in hard-rock tunnelling.

## Discrete element theory

### Discrete element theory

**Fig. 2:**
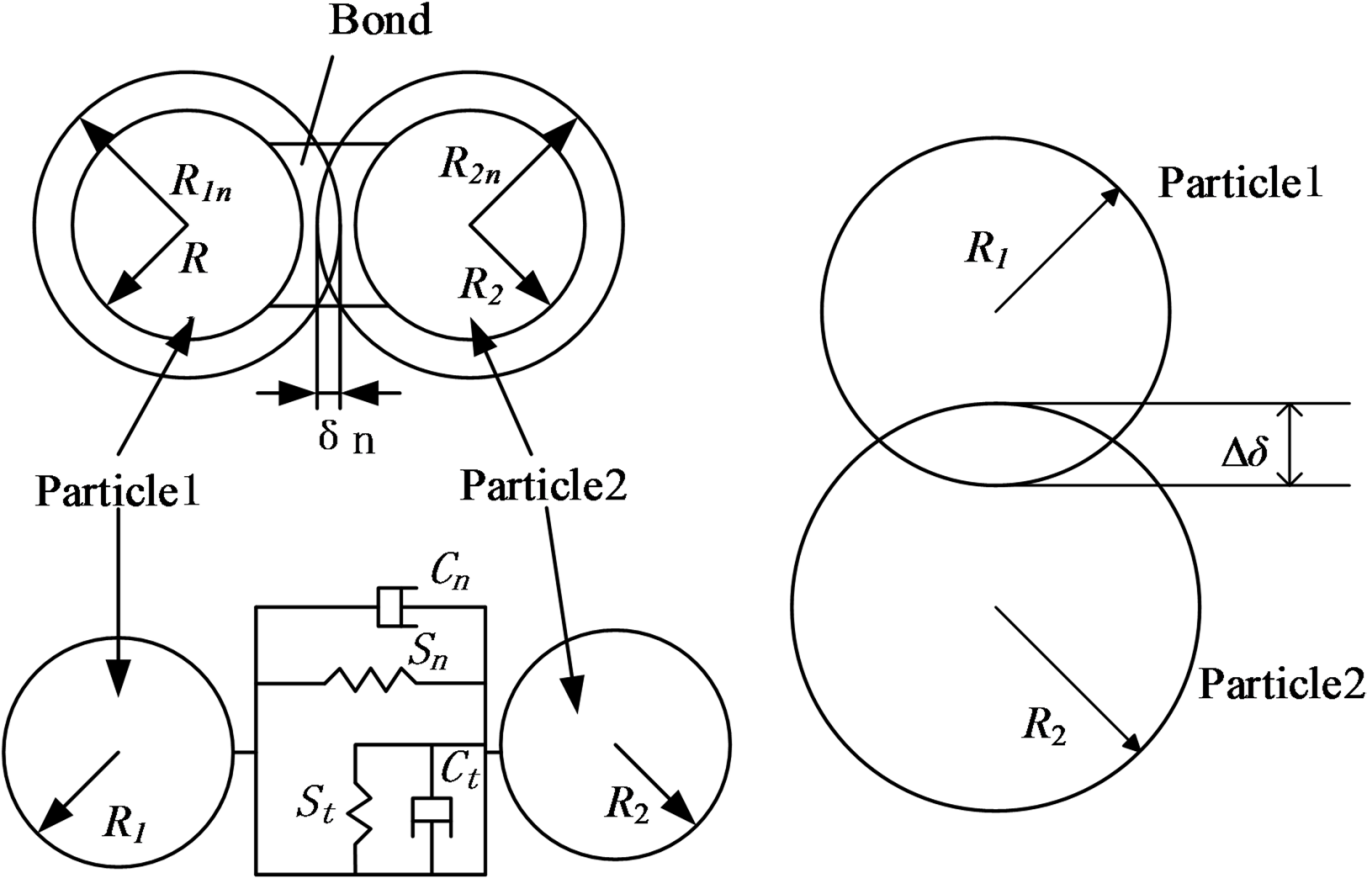
Schematic diagram of the model.

The discrete element method (DEM), which has its origins in molecular dynamics, focuses on the mechanical behaviour of discontinuous media^14^ and can describe rupture and fracture development of particulate assemblages from a microstructural perspective^15^. Common contact models implemented in EDEM include the Hertz–Mindlin adhesive contact model, Hertz–Mindlin with JKR, Hertz–Mindlin (no slip) with heat conduction, and the hysteretic spring model. Because the actual pre-cracking process does not involve significant electrical or thermal interactions between rock particles and the cracking mechanism, the Hertz–Mindlin contact model without adhesion (hereafter referred to as the H–M bonding model) is selected^16,18–20,25^. The H–M bonding model can simulate crack initiation and propagation: when a contact force exceeds the bond strength in any direction, the bond breaks and fracture bonds appear; a subsequent chain reaction produces a crack network that manifests macroscopically as crack extension. Thus, the H–M bonding model can reproduce mechanical behaviours related to crack propagation. The schematic of the basic H–M bonding model is shown in Fig. 2.

The influence of microstructure on macroscopic behaviour can be captured by the H–M bonding model^17^. In this model, the contact transforms into bonded behaviour until the inter-particle force exceeds the bond strength; bond rupture (caused by uplift or truncation) produces artificial cracks. The damage condition is expressed as:

2 $$\left\{ {\begin{array}{*{20}l} {\sigma _{{\max }} < \frac{{ - F_{n} }}{A} + \frac{{2T_{t} }}{J}R} \hfill \\ {\tau _{{\max }} < \frac{{ - F_{t} }}{A} + \frac{{T_{n} }}{J}R} \hfill \\ \end{array} } \right.$$

Style: *A=*π*R*^2^, *A* is the contact area, mm²; *J*=π*R*^4^/2, *J* is the moment of inertia, mm⁴; $R = \sqrt {R_{1} R_{2} }$, *R* is the bonding radius of the particles, mm; *R*_*1*_, *R*_*2*_, are the radii of particles 1 and 2, respectively, mm; *F*_*n*_, *F*_*t*_, are the normal and tangential forces on the particles, respectively, N; *T*_*n*_, *T*_*t*_, are the normal and tangential moments of the particles, respectively, N·m.

### Rock wall modeling

Construction of a virtual rock wall is a prerequisite for subsequent pre-fracture modelling and DEM–MFBD coupling. To ensure the numerical rock wall closely represents field working conditions, the model must approximate the pre-fracture and cutting geometry of the roadheader. A curved rock wall—centered on the slewing table with the cutting arm as radius—was created in SolidWorks and imported into EDEM. Particles with a radius of 12 mm^18,19^ and a bonding radius of 14 mm were generated to build a 1.5 m × 1.0 m × 1.0 m coal-rock model comprising 304,611 particles. The physico-mechanical and bonding parameters of the rock-wall particles are listed in Tables 1 and 2^20^.

**Table 1:** Physical parameters of rock wall particles.

Parameters	Numerical value
Density (kg m^−3^)	2600
Compressive strength (MPa)	71
Young’s modulus (MPa)	21,500
Poisson’s ratio	0.19
Coefficient of restitution	0.65
Coefficient of static friction	0.85
Coefficient of rolling friction	0.35
Coefficient of firmness	8
Angle of internal friction (°)	40.08

**Table 2:** Bonding parameters of rock wall particles.

Parameters	Numerical value
Phase stiffness in the parallel bonding method(N·m^− 3^)	2.3017e + 09
Tangential phase stiffness of parallel bonding(N·m^− 3^)	1.8775e + 09
Maximum stress in the parallel-bonding phase(MPa)	28.936
Maximum tangential stress of parallel bonding(MPa)	12.174

### Mechanical pre-cracking of rock walls

**Fig. 3:**
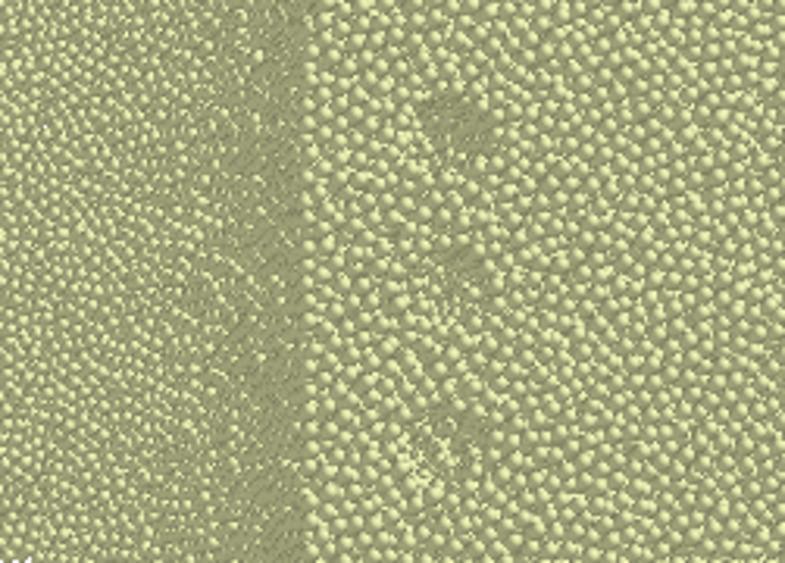
Rock wall model.

The hard rock considered here is relatively intact, with high tensile, compressive and shear strengths and low deformability under external loading^21^. To generate the pre-cracked rock wall, an API was written using Visual Studio; particles were removed to create prefabricated holes. A rock wall containing three pre-cracked holes (diameter 100 mm, depth 500 mm) with a hole spacing of 200 mm was produced, as illustrated in Fig. 3.

The hydraulic riser assembly was modelled in SolidWorks, imported into EDEM, and positioned within the prefabricated hole.

A velocity is assigned to the hydraulic riser in EDEM to simulate the pre-cracking process. In engineering practice, the riser ruptures the rock wall by being driven by a hydraulic cylinder; the riser velocity is provided by hydraulic oil acting on the piston. The hydraulic cylinder propulsion speed is set to 0.5 m·s⁻¹, which complies with specifications for the operating speed of rubber seals^22^, balancing sealing performance and wear control. Accordingly, the riser velocity in this study is set to 0.5 m·s⁻¹.

### Crack formation

**Fig. 4:**
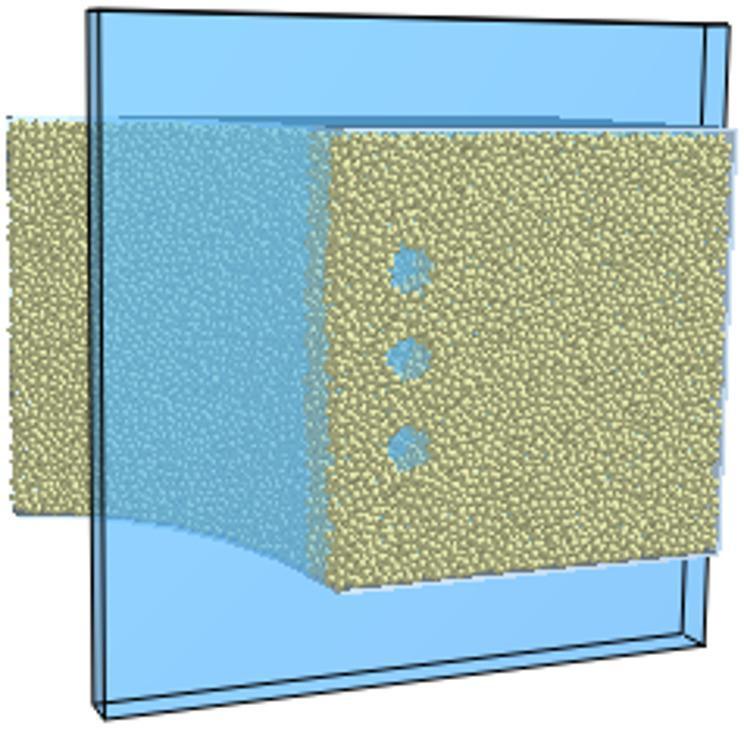
Regional location.

**Fig. 5:**
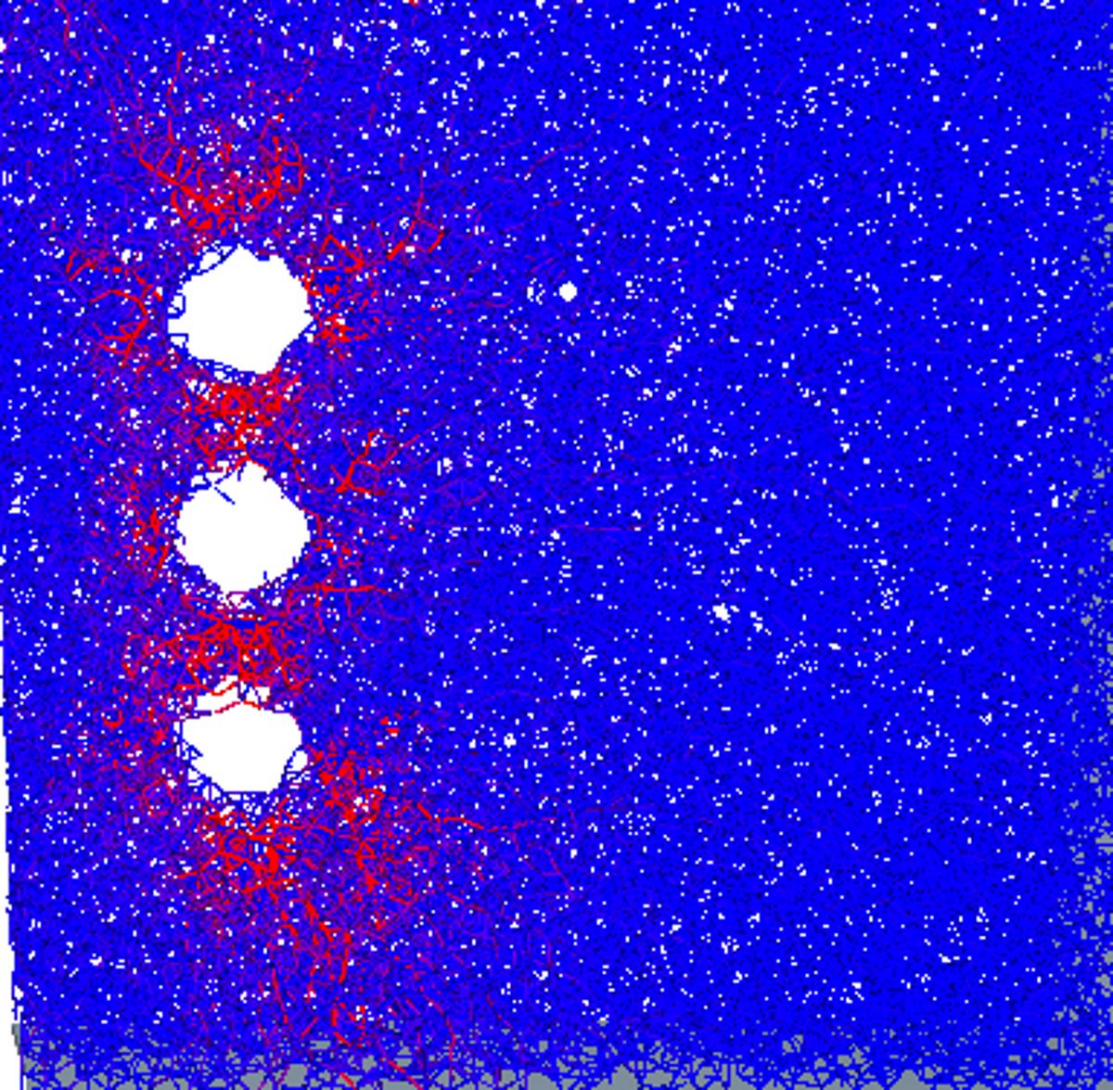
Regional cracks.

To observe the pre-cracking process more clearly, the region shown in Fig. 4—a vertical spatial cross-section—was established in EDEM. Cracks formed in the rock wall after pre-cracking are shown in Fig. 5.

According to the discrete-element bonding model, uplift pressure applied to the rock produces internal cracks, which are represented by fracture bonds between particles. When particles are removed to generate the prefabricated holes, the number of fracture bonds is 1,009; after the pre-cracking operation, the number of fracture bonds increases to 3,618.

## Modeling of rigid–flexible coupling of the roadheader

**Fig. 6:**
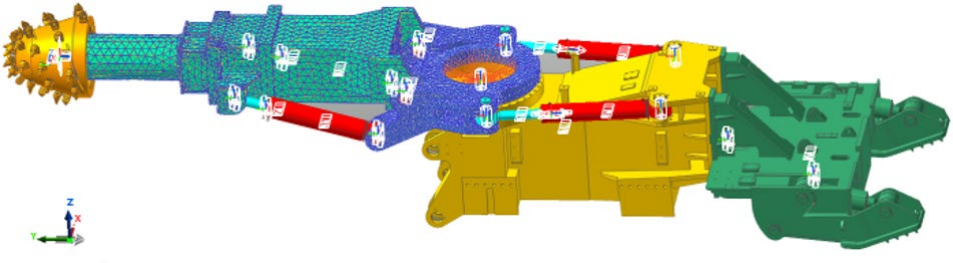
Virtual prototype of the roadheader.

This study examines the EBZ200 roadheader. A three-dimensional solid model of the roadheader was created in SolidWorks and imported into RecurDyn to apply kinematic and boundary constraints. The principal constraints applied are the rotating vice, moving vice and fixed vice, as illustrated in Fig. 6.

### Establishment of flexible model for key parts of the roadheader

The cutter boom and slewing (rotary) table were modelled as flexible bodies using the G-Manager within RecurDyn’s Flexible module. In the Mesher interface, the geometry was refined with Geo.refine; rigid regions were identified with the Assist command; and meshing was completed using the Solid4 (3D tetrahedral) cell type via AutoMesh^23^. Mesh sizing was specified as follows: the cutting-arm minimum element size = 10 mm and maximum = 50 mm; the rotary-table global default mesh size = 25 mm, with local refinement to 15 mm in the region connecting to the cutting arm. To support subsequent vibration analysis, patches were created for the cutting arm and rotary table using the Patch command. The resulting rigid–flexible coupled virtual prototype of the roadheader is shown in Fig. 6.

### DEM–MFBD bidirectional coupling simulation setup

Data exchange between the multibody dynamics software RecurDyn and the discrete-element software EDEM is implemented using wall-format files^24^. During rock cutting, the two packages operate in a bidirectionally coupled mode via the External SPI interface, enabling real-time transfer of contact and cutting information. Material parameters for the roadheader cutter head are listed in Table 3.

**Table 3:** Cut-off head material parameters.

Parameters	Numerical value
Density (kg m⁻³)	7850
Poisson’s ratio	0.31
Shear modulus (MPa)	81,000

### Simulation and result analysis

**Fig. 7:**
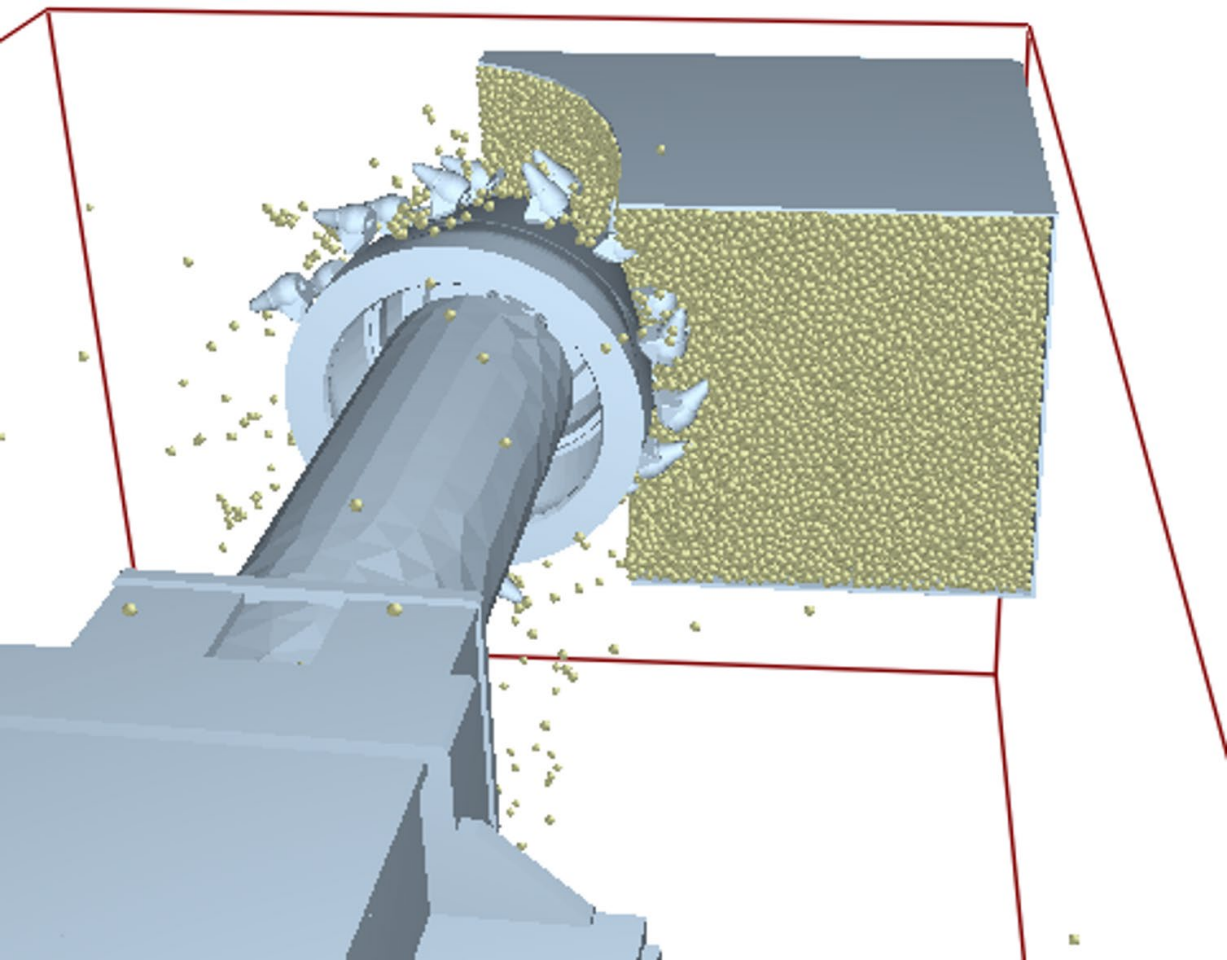
Diagram of the truncation process.

**Fig. 8:**
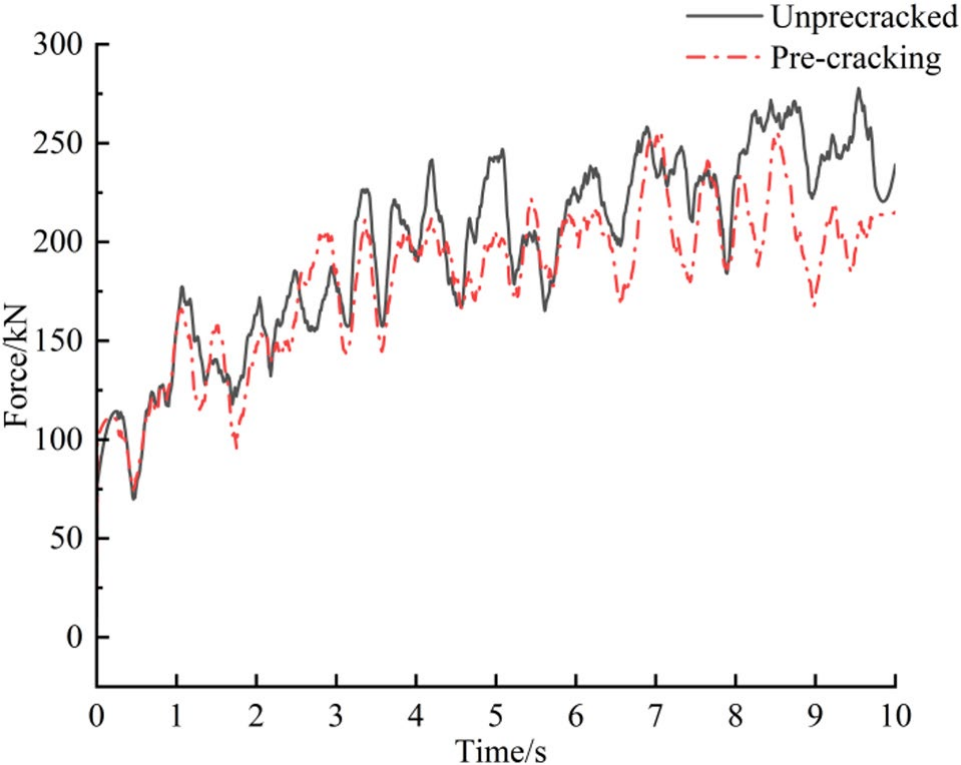
Combined load curve.

**Fig. 9:**
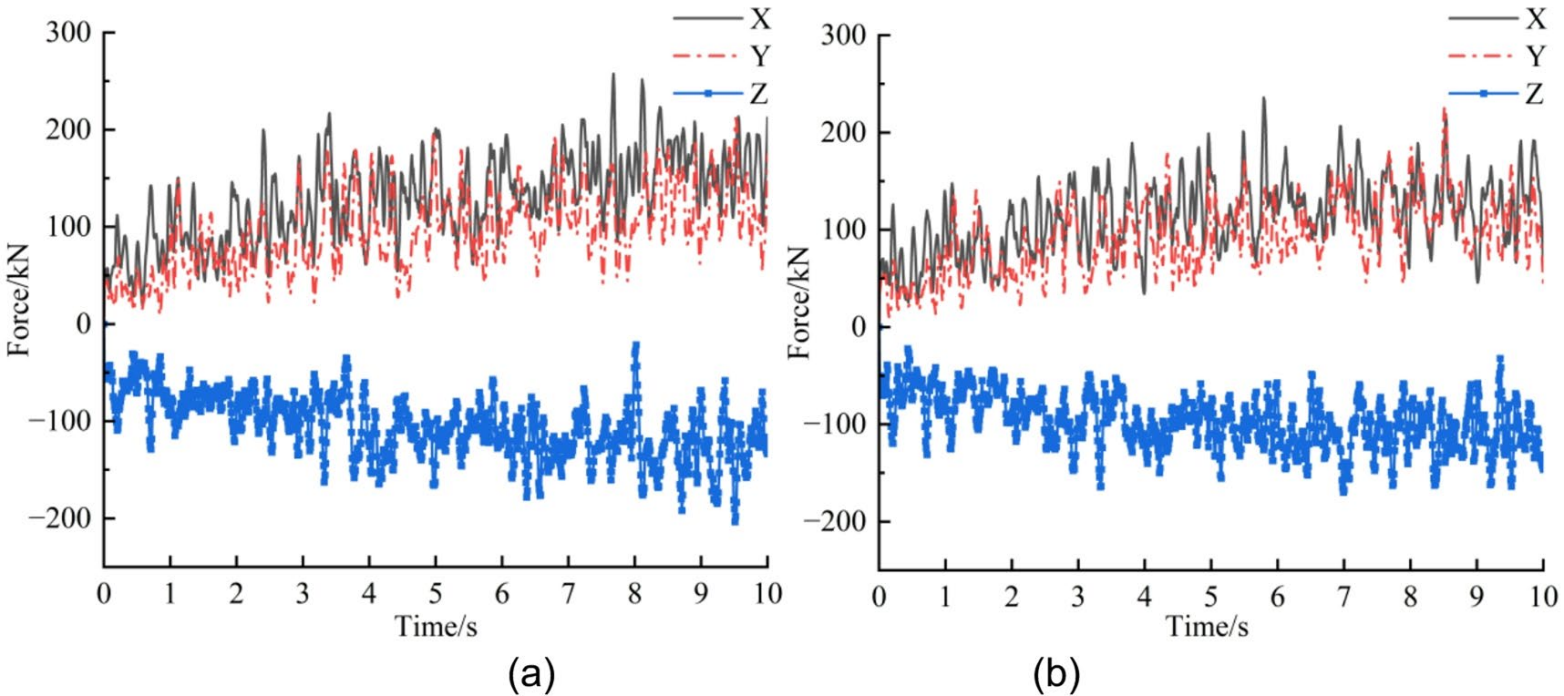
Three-directional force load curves. (**a**) Un-precracked, (**b**) pre-cracked.

In RecurDyn, the traverse speed of the roadheader was set to 1.38 m·min⁻¹ in the negative X direction. The cutting-head rotational speed was set to 45 r·min⁻¹. The total simulation duration was 10 s, with 1,000 time steps; in EDEM the data saving interval was 0.01 s and the coupled simulation was launched. The truncation process of the rock wall by the roadheader is shown in Fig. 7. Post-processing in EDEM produced the resultant (combined) force and three-direction force histories of the cutting head for the un-precracked and pre-cracked rock walls, presented in Figs. 8 and 9, respectively. In this study, the “un-precracked” condition denotes a rock wall cut by the roadheader without prior drilling or pre-cracking.

Analysis of the load histories in Fig. 8 indicates that, from 0 s to 3 s, the cutting head makes initial contact and progressively penetrates the rock wall; the two curves display a similar rising trend in this interval because the cutting head has not yet engaged the prefabricated cracks. From 3 s to 7 s the cutting head begins to interact with the cracks, and the combined load for the pre-cracked case remains lower than that of the un-precracked case. When the cutting head engages the holes more fully (7–10 s), the combined load for the pre-cracked case stabilises and no longer increases. To quantify stability, a load-fluctuation factor was introduced and calculated as^25^:

3 $$\delta = \frac{1}{{\overline{F} }}\sqrt {\frac{{\sum\nolimits_{{i = 1}}^{n} {\left( {F_{i} - \overline{F} } \right)^{2} } }}{n}}$$

4 $$\overline{F} = \frac{1}{n}\sum\limits_{{i = 1}}^{n} {F_{i} }$$

Style:*F*_*i*_ is the instantaneous value of the cutting-head load; $\overline{F}$ is the mean value of the cutting-head load; *n* is the number of statistical samples.

The load-fluctuation coefficient—defined as the ratio of the standard deviation to the mean—is adopted to assess the stability of the cutting load. As a dimensionless metric, it removes the influence of differing mean loads and thus more objectively characterises relative fluctuations under different working conditions. In engineering practice, this coefficient reflects dynamic load fluctuation of the system and its effect on roadheader vibration; therefore, analysing the cutting-head load-fluctuation coefficient has clear engineering significance.

The average resultant load on the cutting head after pre-cracking is 181.56 kN, compared with 197.67 kN for the un-precracked case — an 8.2% reduction. The load-fluctuation coefficient decreases from 0.0242 (un-precracked) to 0.0213 (pre-cracked), which is attributable to reduction of the effective bonding stiffness within the rock mass caused by mechanical pre-cracking.

Figure 9 shows the three-directional force histories. When the cutting head penetrates the rock wall, forces increase in all three axes. Fluctuation of the lateral component (Y direction, cutting-depth direction) is relatively small. In the traction direction (X), the traction resistance is larger than the cutting resistance in the Z direction. After pre-cracking, the upward trends of the X- and Z-direction curves are markedly attenuated once the head cuts into the cracks, whereas the Y-direction curve exhibits little change. Quantitatively, the mean X, Y and Z loads of the cutting head in the un-precracked case are 129.3 kN, 94.85 kN and − 105.1 kN, respectively; after pre-cracking the corresponding means are 114.2 kN, 91.54 kN and − 97.0 kN. Thus, average loads in the X, Y and Z directions decrease by 11.7%, 3.5% and 7.7%, respectively, following pre-cracking.

## Vibration characterization of the roadheader

**Fig. 10:**
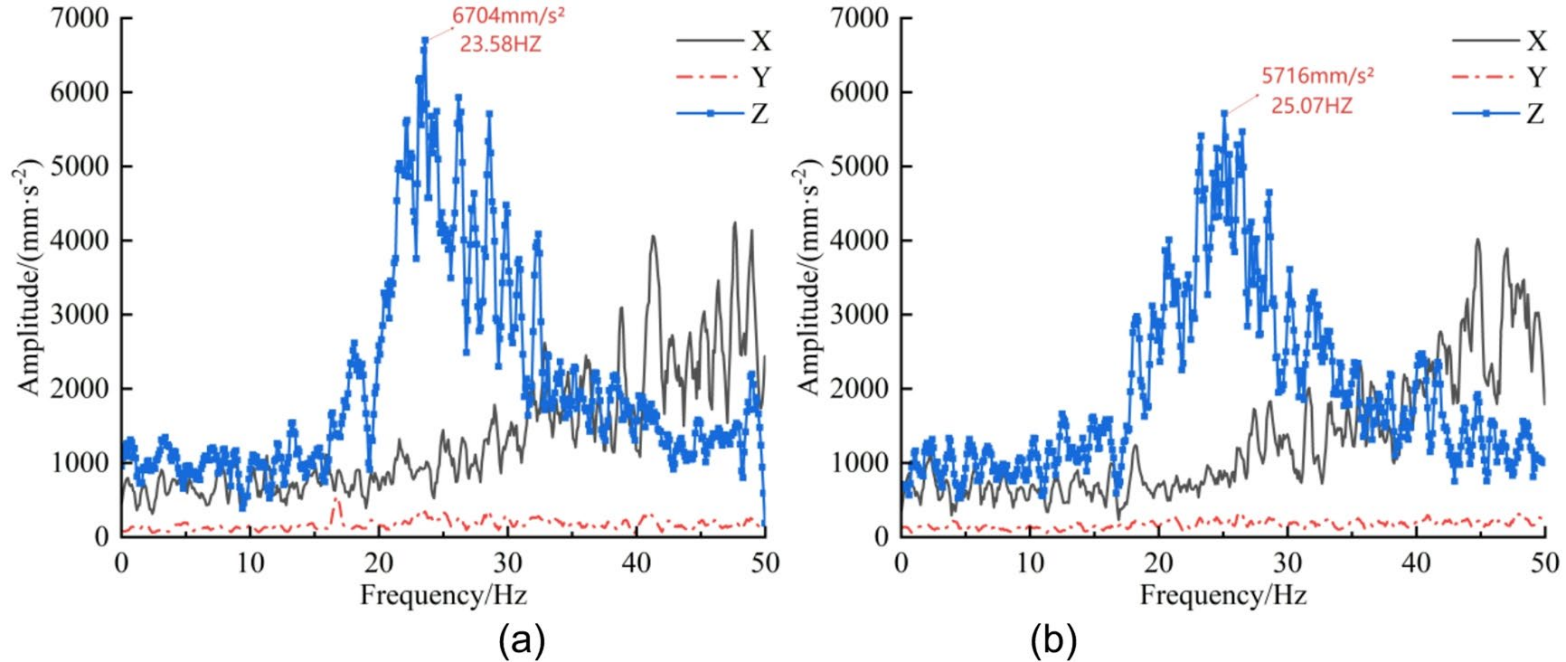
Cut-off head three-axis acceleration—frequency-domain curves. (**a**) Un-precracked, (**b**) Pre-cracked.

**Fig. 11:**
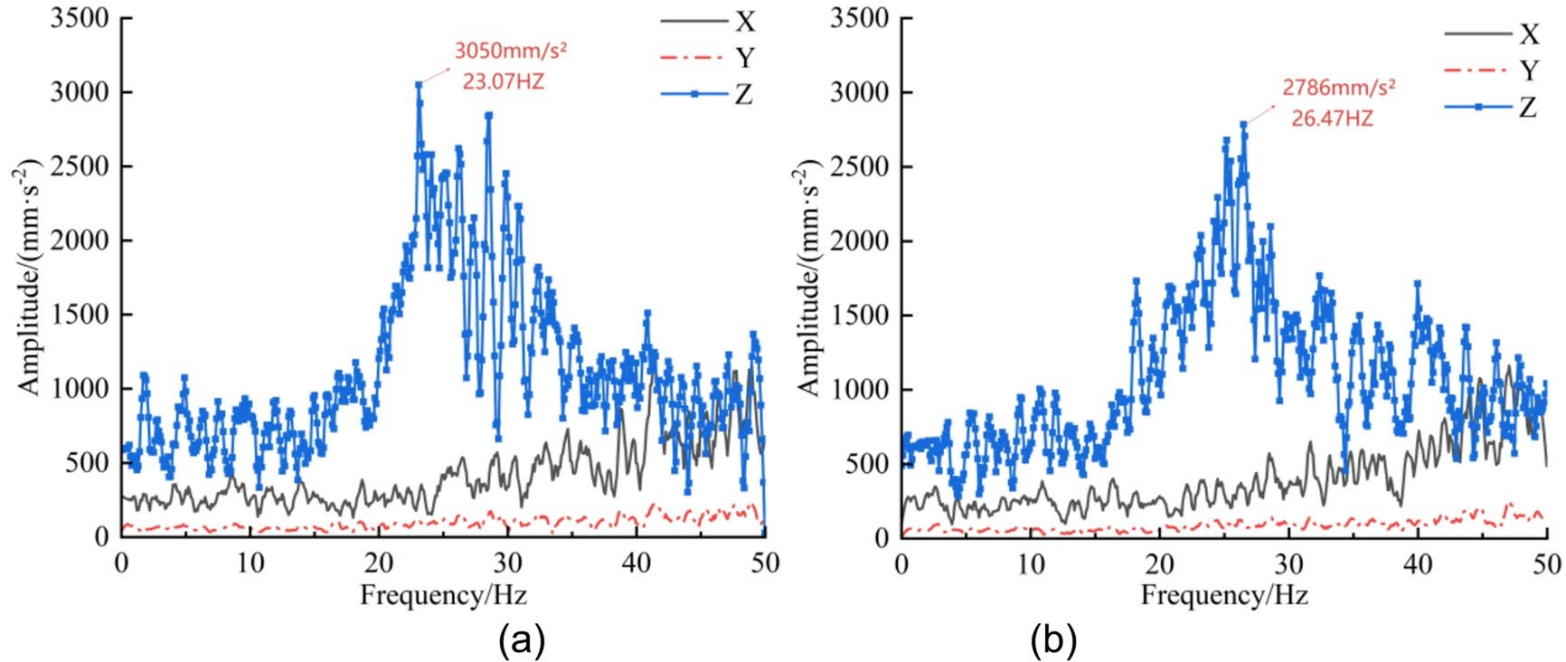
Cut-off arm three-axis acceleration—frequency-domain curves. (**a**) Un-precracked, (**b**) Pre-cracked.

**Fig. 12:**
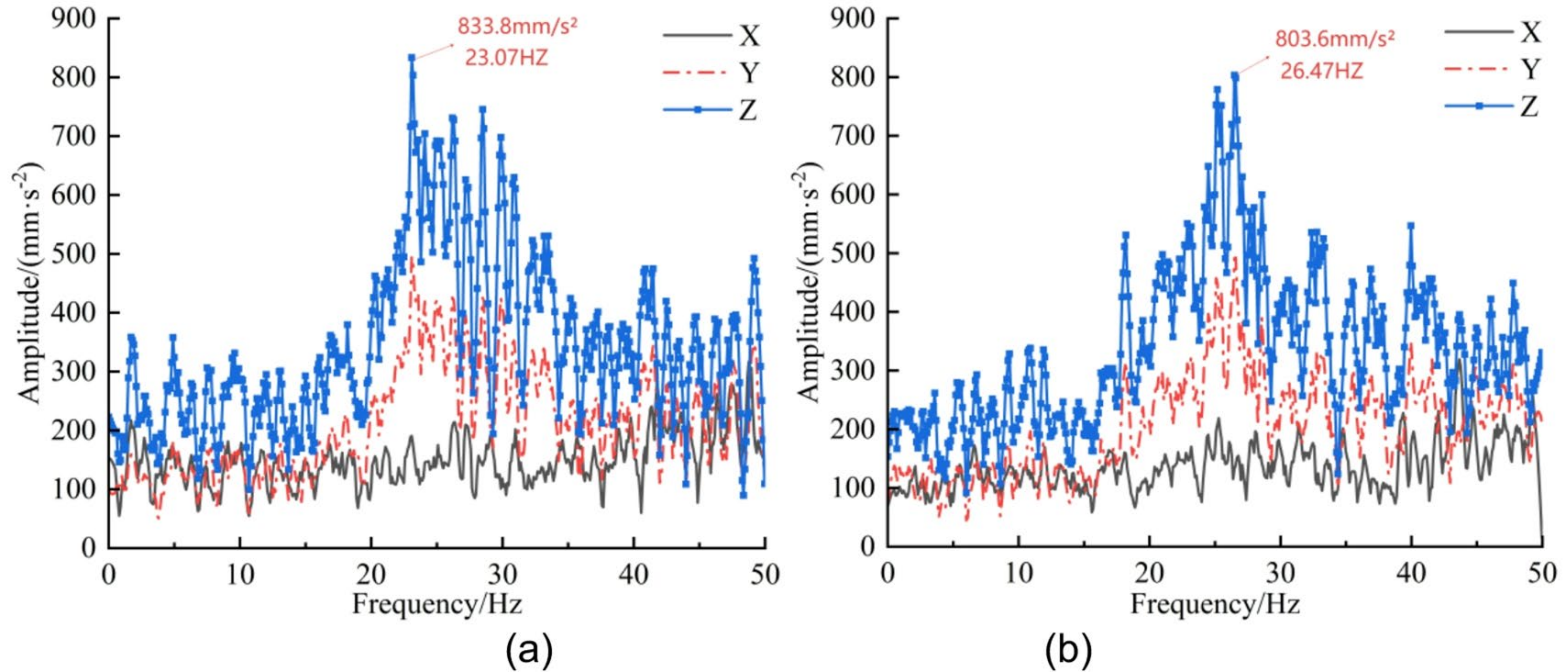
Rotary table three-axis acceleration—frequency-domain curves. (**a**) Un-precracked, (**b**) Pre-cracked.

Through the bidirectional coupling simulation between EDEM and RecurDyn, three-axis vibration accelerations of the cut-off head, cut-off arm, and rotary table were extracted by post-processing in RecurDyn. The signals were plotted using Origin and the time-domain responses were transformed into the frequency domain via Fourier transform^26^, as shown in Figs. 10, 11 and 12.

Analysis of the cut-off-head frequency spectra in Fig. 10 indicates a dominant vibration band at 20–30 Hz for both the un-precracked and pre-cracked rock walls. In this band, the Z-direction amplitude for the un-precracked case attains a peak of 6704 mm·s⁻² at 23.58 Hz, whereas the pre-cracked case reaches a peak of 5716 mm·s⁻² at 25.07 Hz, representing a 14.7% reduction after pre-cracking. Further inspection of Figs. 11 and 12 shows that the cut-off arm and the rotary table also display pronounced vibration in the 20–30 Hz range for both cases. The cut-off-arm peak amplitude for the un-precracked case is 3050 mm·s⁻² at 23.07 Hz, while the pre-cracked peak is 2786 mm·s⁻² at 26.47 Hz (an 8.7% decrease). The rotary-table peak reduces from 833.8 mm·s⁻² at 23.07 Hz to 803.6 mm·s⁻² after pre-cracking, a 3.6% decrease. Although the overall spectral shapes of the pre-cracked and un-precracked responses in the X, Y and Z directions are similar, amplitudes are substantially reduced in certain frequency bands following pre-cracking. The attenuation sequence of components follows the law “cut-off head > cut-off arm > rotary table”, and the principal vibration frequencies exhibit an upward shift of approximately 2–3 Hz. This behaviour arises because mechanical pre-cracking alters the dynamic characteristics of the rock mass: it reduces the effective stiffness of the rock and modifies the rock-breaking mechanism, thereby lowering vibration intensity and changing the system’s dynamic response.

From the frequency-domain results it is evident that, for both pre-cracked and un-precracked conditions, the ordering of vibration-acceleration magnitudes for the cut-off head and cut-off arm is Z > X > Y, whereas for the rotary table the ordering is Z > Y > X. Overall, the reduction in vibration acceleration across the three components follows the same hierarchy: cut-off head > cut-off arm > rotary table.

The formation of crack slits by mechanical pre-cracking modifies the dynamic interaction between the rock mass and the roadheader. Crack propagation substantially weakens the macroscopic stiffness of the rock mass so that the fracture process evolves from abrupt, system-scale failure toward progressive, localized damage. This transition effectively reduces the impact characteristics and the fluctuation amplitude of the cutting load. Concurrently, the crack network introduces additional pathways for energy dissipation, markedly reducing the vibration energy transmitted to the mechanical structure. The combined effects of source weakening and energy redistribution produce a clear attenuation of the structural vibration response along the transmission path, ordered from cut-off head → cut-off arm → rotary table.

## Roadheader cutting hard-rock vibration test

**Fig. 13:**
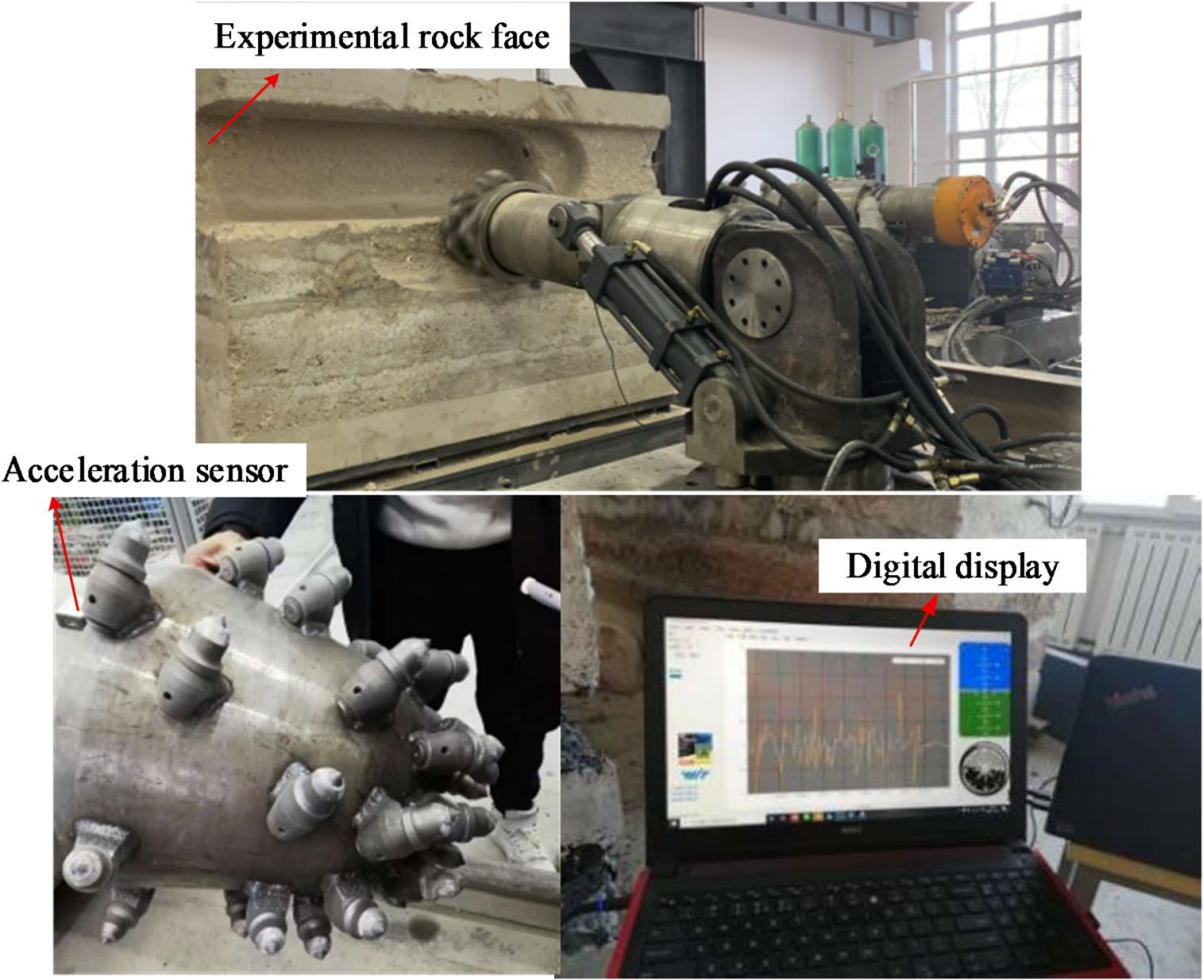
Roadheader vibration field test layout.

To verify the numerical model, this study adopts cross-validation between field testing and simulation. Field tests used an EBZ200 roadheader platform: the cutting-head rated power is 200 kW and the cutting-head rotational speed is 45 r·min⁻¹; the cutting picks are arranged uniformly along a specified helix. Test rock was sampled on site and identified as siltstone, with an average Mohs hardness of 8 and an approximate uniaxial compressive strength of 30 MPa. Vibration was measured using PCB Piezotronics 356A16 three-axis ICP accelerometers, which were fixed to the cutting-head connecting flange and the cantilever via magnetic bases for real-time acquisition^27^; signals were recorded by a DH5922D dynamic data acquisition system. Pre-cracking holes were produced using an SCZ-100 hydraulic drilling rig with a φ100 mm diamond bit to a depth of 500 mm and spacing of 200 mm, consistent with the simulation parameters. The field test layout is shown in Fig. 13.

**Fig. 14:**
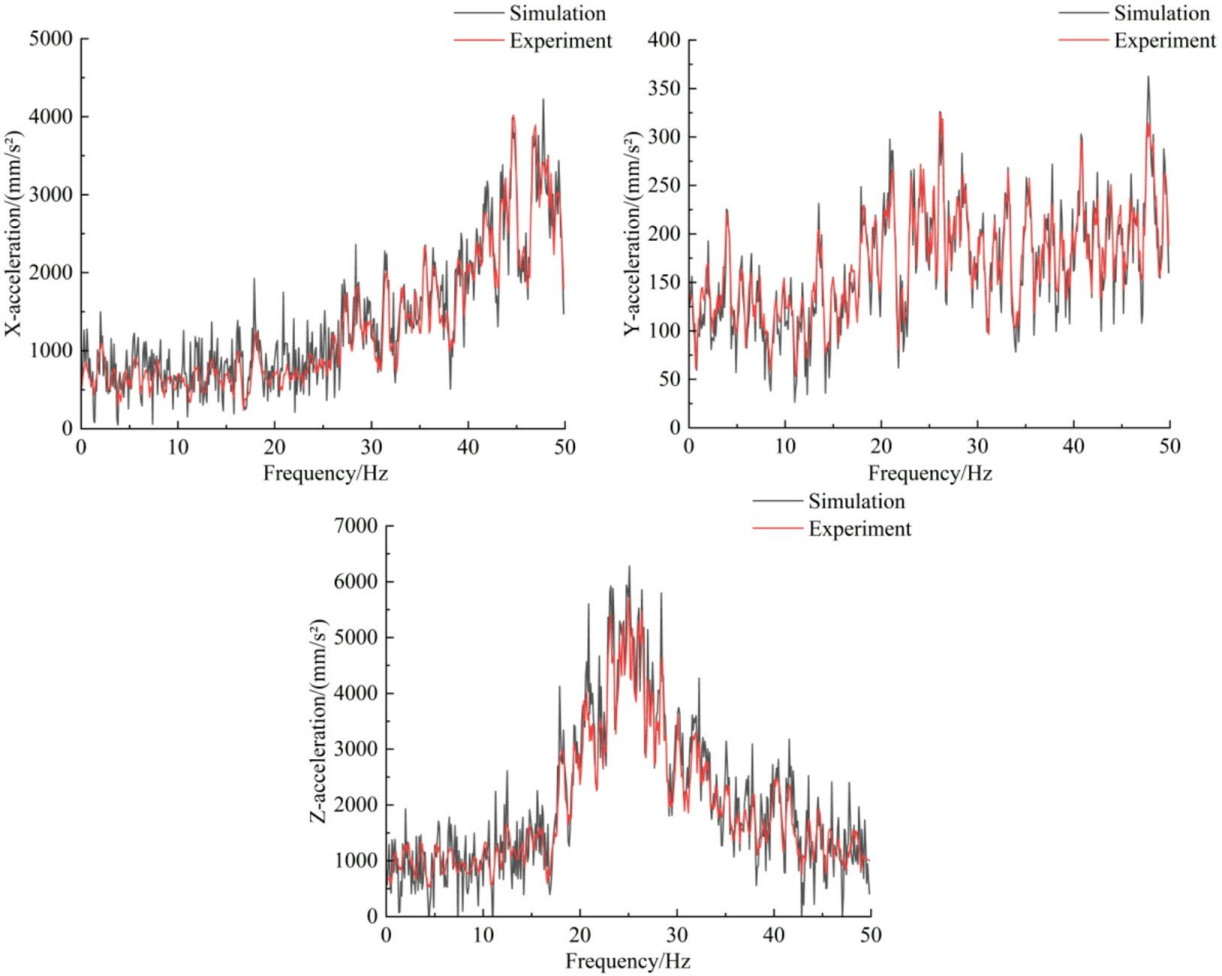
Frequency-domain acceleration comparison curve.

Test data were exported and compared with simulation results; Fig. 14 presents the frequency-domain acceleration comparison. The simulated and measured three-axis cutting-head acceleration trends agree closely. Under identical operating parameters, the time-domain Z-direction acceleration signals from test and simulation exhibit consistent waveform characteristics, principal vibration bands and amplitude ranges. The correlation coefficient between measured and simulated Z-direction signals is 0.95, indicating a strong linear relationship and high consistency in trend. The test also confirms that Z-direction acceleration of the cutting head is markedly greater than X- and Y-direction accelerations — a feature reproduced by the numerical model.

The above comparative validation demonstrates that the developed DEM–MFBD coupled model effectively captures the dynamic behaviour of the actual cutting system. On the basis of this validation, subsequent vibration analyses of roadheader cutting into pre-cracked rock using this model are considered credible.

## Conclusions

Under the specific test conditions adopted in this study (rock wall: siltstone, mean Mohs hardness = 8, uniaxial compressive strength ≈ 30 MPa; pre-cracking parameters: three pre-crack holes, Ø100 mm, depth 500 mm, spacing 200 mm, riser velocity 0.5 m·s⁻¹; cutting parameters: EBZ200 cutting-head rotational speed 45 r·min⁻¹, traverse swing speed 1.38 m·min⁻¹; simulation duration = 10 s), the rock wall was mechanically pre-cracked and the following conclusions were obtained from DEM–MFBD two-way coupled simulations of the roadheader cutting process:

Mechanical pre-cracking realizes vibration attenuation by weakening the rock mass at the source of excitation. The results indicate that the crack gaps generated by pre-cracking substantially reduce the effective stiffness of the rock mass, producing an 8.2% reduction in the mean resultant load of the cutting head, and reductions of 11.7%, 3.5% and 7.7% in the X, Y and Z directional mean loads, respectively. Concurrently, the load-fluctuation coefficient decreases from 0.0242 to 0.0213. These changes correspond to a transformation of the rock-breakage mode from violent “overall brittle fracture” to more gradual, localized crushing along crack gaps, thereby fundamentally mitigating the strength of the vibration excitation source.

A gradient attenuation law of structural vibration response along the transmission path is revealed. The cutting head, cutting arm and rotary table all exhibit strong vibration in the 20–30 Hz band when cutting both pre-cracked and un-precracked rock walls. Compared with the un-precracked case, the maximal vibration-acceleration magnitude in the frequency domain is reduced by 14.7% for the cutting head, 8.7% for the cutting arm and 3.6% for the rotary table when cutting the pre-cracked rock wall. Consequently, vibration intensity decays progressively along the transmission chain “cutting head → cutting arm → rotary table”. These findings demonstrate that mechanical pre-cracking not only attenuates the excitation source but also optimises vibration transmission within the mechanical structure by modifying the rock–machine interaction, thereby affording protection to key components.

The study provides direct, practical guidance for engineering application. The pre-cracking parameters validated here (hole diameter 100 mm, hole depth 500 mm, hole spacing 200 mm) offer an actionable basis for active rock-structure design to control vibration in field operations. The results show that optimizing pre-cracking schemes can effectively reduce dynamic equipment loads, which is of considerable significance for enhancing roadheader operational reliability and extending the service life of critical components.

The present study has several limitations. Although the 10-s simulation (corresponding to seven revolutions of the cutting head) is sufficient to capture the dynamic characteristics of the cutting phase, tool wear is a cumulative process occurring over prolonged operation; the effects of cutting-tooth wear on cutting forces and roadheader vibration were not considered. In addition, only a limited number of field tests (single validation per working condition) were conducted, which constrains comprehensive assessment of model applicability across diverse geological settings. These limitations may reduce the generalisability of the findings for long-term continuous operation and complex lithologies. Future work should extend simulation durations toward full operating-cycle orders, increase the number of experimental replicates across multiple rock types, and implement statistical significance testing to strengthen the robustness and reliability of the results.

## Supplementary Information

Below is the link to the electronic supplementary material.

Supplementary Material 1

## Data Availability

The datasets obtained from the analysis or the field measurement in this study are available through the corresponding author on the reasonable requirement.
